# The Role of ATP-Binding Cassette Subfamily A in Colorectal Cancer Progression and Resistance

**DOI:** 10.3390/ijms24021344

**Published:** 2023-01-10

**Authors:** Latifa Alketbi, Abeer Al-Ali, Iman M. Talaat, Qutayba Hamid, Khuloud Bajbouj

**Affiliations:** 1 College of Medicine, University of Sharjah, Sharjah P.O. Box 27272, United Arab Emirates; 2 Sharjah Institute for Medical Research, University of Sharjah, Sharjah P.O. Box 27272, United Arab Emirates; 3 Faculty of Medicine, Alexandria University, Alexandria 21544, Egypt; 4 Meakins-Christie Laboratories, McGill University, Montreal, QC H3A 0G4, Canada

**Keywords:** colorectal cancer, ATP-binding cassette, ATP-binding cassette subfamily A, chemoresistance

## Abstract

Colorectal cancer (CRC) is one of the most common malignancies worldwide; it is the fourth leading cause of cancer-related deaths. CRC arises due to mutations that can affect oncogenes, tumour suppressor genes and DNA repair genes. The lack of novel diagnostic and therapeutic targets and the development of chemoresistance are some of the major issues when dealing with CRC. The overexpression of ATP-binding cassette (ABC) transporters is considered one facilitating mechanism for chemoresistance. Furthermore, ABC transporters have additional roles in cancer development beyond multidrug resistance. In CRC, lipid dysregulation has a key role in tumour development and progression, as cancer cells rely on lipids for energy and rapid cell proliferation. ABC subfamily A (ABCA) contains the largest members of ABC proteins, mainly known for their role in lipid transport, mostly membrane lipids such as cholesterol and phospholipids. Although the exact mechanism of action of these members is not confirmed, their expression is usually correlated with tumour progression and therapy resistance, probably due to their role in lipid homeostasis. CRC shows alteration in the expression of ABCA transporters, which is usually linked to poor prognosis and overall survival. Therefore, as lipid transporters, their role in CRC is investigated, and their diagnostic and prognostic potential is evaluated. This minireview presents evidence from various studies suggesting that ABCA transporters might have an active role in CRC and can be utilized as potential diagnostic and therapeutic targets.

## 1. Introduction

Colorectal cancer (CRC) is the third most common cancer worldwide and the fourth leading cause of cancer-related deaths. Approximately two million cases occur each year with a five-year survival rate, resulting in nearly 800,000 deaths [1]. CRC, like many other malignancies, can develop due to mutations in either oncogenes, tumour suppressor genes or genes involved in DNA repair, and it is classified based on the origin of the mutation. Mutations in numerous molecular pathways are involved in CRC development. The first example is the loss of tumour suppressor genes such as Tumour protein (p53) and Adenomatous polyposis coli (APC); their deletion in CRC promotes tumour progression and development [2]. Second, a mutation in the oncogene Kirsten rat sarcoma (KRAS) is frequently detected in CRC with 40% of patients having a missense mutation in the KRAS gene resulting in its activation which leads to an increase in cellular growth and proliferation [2,3]. Along with KRAS, BRAF can code for proteins that result in unregulated activation of signalling pathways such as Ras-Raf-MEK-ERK resulting in cellular growth and proliferation [2]. In addition, a mutation in PIK3CA and SMAD4 can also lead to an increase in the risk of metastasis and invasiveness of the tumour. These genetic alterations that can be observed in CRC promote the activation of signalling pathways that are involved in survival, cell proliferation and cell-cycle progression; the two main pathways that are altered in CRC are Epidermal Growth Factor Receptor-Reticular Activating System (EGFR-RAS) and Wnt-β-catenin pathways [4]. Most CRC cases develop sporadically and slowly over the years. The rest are hereditary, as in hereditary nonpolyposis colorectal cancer (HNPCC) and familial adenomatous polyposis (FAP) and some forms of long-standing inflammatory bowel diseases [5]. The most common histologic type of CRC is adenocarcinoma (>90%) which can be classified into well, moderately and poorly differentiated adenocarcinoma; other rare types include mucinous carcinoma, squamous cell carcinoma, adenosquamous and undifferentiated carcinoma [6]. Treatment for CRC depends on whether it is metastasised or not; when it comes to primary nonmetastatic CRC, it is mainly treated by surgery with complete mesocolic excision (CME) [7]. On the other hand, despite the breakthroughs in therapy and multidisciplinary care, there is no cure for metastatic CRC (mCRC); therefore, chemotherapy using a combination of chemotherapeutic drugs such as FOLFOX (5-FU, oxaliplatin and leucovorin) or FOLFIRI (5-FU, irinotecan and leucovorin) and targeted therapy in which it targets a specific pathway or feature in cancer, for instance blocking EGFR or vascular endothelial growth factor signalling, is currently used for patients with mCRC [8,9]. A multikinase inhibitor such as Regorafenib (REG) is also used to treat mCRC. REG acts by blocking the activity of different protein kinases that facilitate tumour proliferation and angiogenesis [10]. As described earlier, mutation due to defects in DNA repair can give rise to malignancies including CRC, where mismatch-repair deficiency (dMMR) is found in approximately 15% of CRC patients which results in tumours high in microsatellite instability (MSI-H) [8]. Interestingly, targeting MSI-H-dMMR using Programmed death 1 (PD-1) inhibitors, namely pembrolizumab, is a promising way to treat CRC compared to chemotherapy, as stated in research conducted by André et al. [8]. When used as first-line therapy for MSI-H-dMMR metastatic colorectal cancer, pembrolizumab resulted in significantly longer progression-free survival than chemotherapy with fewer treatment-related adverse events [8]. Even with the advancement in drug development, therapy resistance remains a major complication when tackling CRC; therefore, finding novel targets is a fundamental approach to overcoming tumour resistance and progression.

ATP-binding cassette (ABC) transporters are the largest protein family, with 48 genes divided into seven subfamilies ranging from ABCA to ABCG. The transporters share common structural properties; they are identified by the presence of two nucleotide-binding domains (NBDs) that bind and hydrolyze ATP and two transmembrane domains (TMDs) that bind to the substrate. The hydrolysis of ATP leads to conformational changes in the NBDs and eventually in the TMDs, which then can bind to their specific substrate and promote its translocation across the cellular membrane [11]. These types of proteins are located on the plasma membrane or the membrane of intracellular compartments such as the lysosome, endosome and endoplasmic reticulum where they function in an energy-dependent mechanism to transport metabolites, hormones, lipids, signalling molecules and drugs across the cellular membrane. Deregulated expression of ABC transporter is very common in malignancies, where they can be over or under-expressed; they are mostly known for their multidrug resistance and promoting therapy resistance in cancer, which is a major obstacle when dealing with cancer therapy. ABC transporters facilitate chemoresistance by promoting the efflux of the drugs outside the cells and reducing the concentration of the drug inside the cells. Well-studied members that are involved in resistance include P-glycoprotein ABCB1 (MDR1), multidrug resistance protein ABCC1 (MRP1) and breast cancer resistance protein ABCG2 (BRCP). Recently many studies revealed the role of ABC transporters in cancer beyond multidrug resistance where they can promote tumour progression and metastasis. This depends on their ability to transport numerous molecules and the activation of pathways that are involved in tumourigenesis [11].

In this review, we will first give an overview of the role of membrane lipids in promoting colorectal cancer initiation and progression, as well as the ATP-binding cassette subfamily A (ABCA) and their function. We will also discuss the possible role of the ABCA subfamily members in CRC progression and therapy resistance.

## 2. The Role of Membrane Lipids in Colorectal Cancer

Lipids are the major structural components of the membrane, and the various types of lipids found are glycerophospholipids, sphingolipids and sterols, mainly cholesterol. They have an important role in determining the physical properties of the membrane and regulating the activity of membrane-bound proteins and they can also function in several signalling reactions by acting as first or second messengers [12]. Metabolic reprogramming is the ability of cancer cells to reprogram their metabolism to meet the increased energy demand caused by continuous growth and rapid cell proliferation, hence the lipid profile in cancer is different from normal tissues due to the alteration in the metabolic processes that are involved in lipid regulation and synthesis [13]. Highly proliferating cancer cells require lipids not only for the formation of the membrane but also for energy metabolism and are used as signalling molecules. Membrane lipids can promote tumourigenesis by the formation of second messengers such as diacylglycerol, phosphatidic acid, lysophosphatidic acid and arachidonic acid from phospholipase-dependent hydrolyzation; these second messengers can activate several signalling cascades including phosphoinositide 3-kinase (PI3Ks), RAC, RAS, RHO and protein kinase C [14]. Cancer cells also show strong avidity for cholesterol; it can act as a regulatory element for sterol regulatory element (SRE)–binding protein (SREBP) activation which regulates lipid biosynthesis and adipogenesis [14,15]. In addition, cholesterol can activate oncogenic smoothened protein (Smo), which plays a role in the hedgehog (Hh) signalling pathway, a pathway that is critical for embryonic development and tumourigenesis [16]. Moreover, lipids also can affect the activity of ABC transporters involved in multidrug resistance (MDR). Intriguingly, cholesterol and phospholipids may be distributed around some MDR transporters such as P-glycoprotein (P-gp) with the high cholesterol levels within the lipid raft supporting the activity of P-gp. It was observed that doxorubicin-resistant CRC cells HT29 expressing P-gp present a higher cholesterol and phospholipids level in lipid rafts compared to drug-sensitive cells [17].

In CRC, dysregulation of the lipid profile and the metabolism has been observed and these alterations can change tumour behaviour affecting its proliferation and survival [18]. These dysregulations mainly involve the membrane lipids; for instance, patients with distant CRC metastases display a higher level of low-density lipoprotein cholesterol, while the level of high-density lipoprotein cholesterol was observed to be higher in low-risk patients [19,20]. A dysregulated level of phospholipids is also correlated with tumour proliferation and development, and the level of phosphatidylcholine is elevated in CRC [21]. Lastly, sphingolipids have a role in cell growth, differentiation and migration; therefore, their dysregulation may influence CRC development [18,22]. A recent study showed that CRC had lower triacylglycerol (TG) and a higher level of membrane lipids, including phospholipids, sphingomyelin and cholesterol; furthermore, the level of saturated fatty acids and polyunsaturated fatty acids were elevated. This study concluded that these lipid composition alterations are associated with increased use of TG for energy and enhanced synthesis of membrane lipids that are crucial for rapid cell proliferation and development [23].

Another study that investigated the lipid composition of different CRC cell lines reported that phospholipid content in tumour cells was higher than in non-tumour cells; in addition, they stated that phospholipid classes, triacylglycerols (TAGs) and cholesteryl-esters (CholE) levels were significantly increased in metastatic SW620 cells compared to primary colon cancer cell line SW480 [24].

## 3. ATP-Binding Cassette Subfamily A

**Figure 1:**
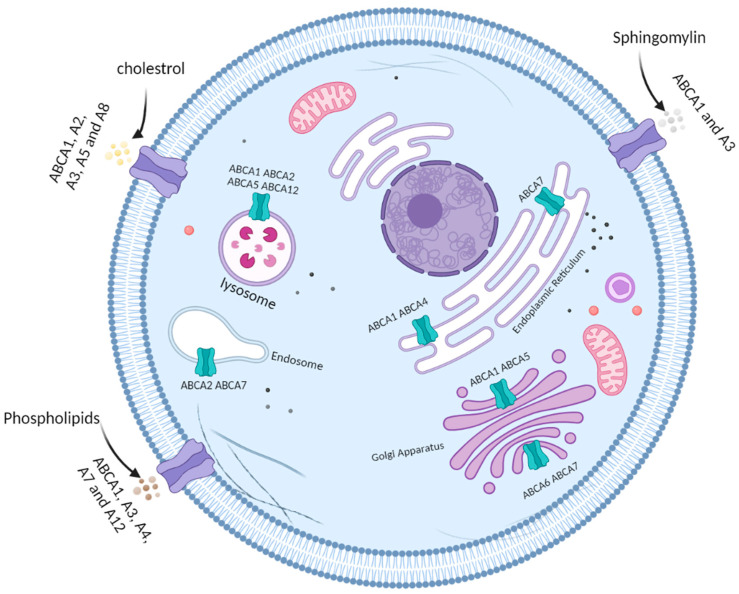
Schematic representation of ABCA subfamily localization and their possible lipophilic substrates. ABCA transporters are localized in the plasma membrane of the cells or in the membrane of specific intracellular organelles such as the lysosome, endosome, endoplasmic reticulum and the Golgi apparatus. Twelve members of the ABCA subfamily of transporters abbreviated ABCA1 through ABCA13 enable the transfer of lipids. ABCA 1, 2, 3, 5 and 8 modulate cholesterol transport. ABCA 1, 3, 4, 7 and 12 transport phospholipids, while ABCA 1 and 3 control the movement of sphingomyelins. Figure generated by BioRender.

ATP-binding cassette subfamily A (ABCA) is a member of ABC transporters; it consists of the largest ABC proteins with a molecular weight of more than 200 kDa [25]. The subfamily consists of 13 members, and they are divided into two groups based on their chromosomal location. Members of the first group are dispersed on six different chromosomes and this includes ABCA1, 2, 3, 4, 7, 12 and 13, whereas the second group is located on chromosome 17 and includes ABCA5, 6,8, 9 and 10; the last transporter ABCA11 is considered a non-functional human gene. Like other ABC transporters, the ABCA subfamily structure consists of two transmembrane domains and two nucleotide-binding domains. Furthermore, they are characterized by the presence of two large extracellular domains [26,27]. ABCA subfamily expression is regulated by a liver X receptor (LXR) transcription factor and sterol regulatory element-binding protein2 (SREBP2), the latter being involved in regulating the cholesterol biosynthesis pathway [28,29]. The mRNA expression of these transporters is found to be lower than their protein expression; this could be explained by the posttranscriptional regulation of ABCA by microRNAs (miRNAs) which can result in upregulation or downregulation in their expression [27,30]. Furthermore, ABCA transporters can be regulated at the posttranslational levels. An example of that would be ABCA1, where the binding to Apo-1 can provide protection from proteolysis; on the other hand, binding to caveolin-1 causes its degradation [31,32]. Different members of the subfamily are distributed differently in the body, which may reflect the various functions of each transporter [27]. They are largely known to be involved in the maintenance of lipid homeostasis; the proteins are localized on the plasma membrane or different cellular compartments such as the lysosome and endosome as indicated in (Figure 1) and are responsible for lipid trafficking and homeostasis. They are responsible for the transport mostly of cholesterol, but also phospholipids and sphingomyelin, and mutation in these transporters usually results in severe diseases related to altered lipid transport [27,33]. In addition, the lipophilic substrates of some members are mentioned; these include phospholipids (for A1, A3, A4, A7 and A12), sphingomyelin (A1 and A3) and cholesterol (transported by A1, A2 and A5) [34]. Moreover, ABCA members are involved in other functions such as monocyte differentiation (ABCA9), macrophage lipid homeostasis (ABCA10), phagocytosis of macrophages (ABCA7) and neurodevelopment in the central nervous system (ABCA13) [27]. Given the diversity in their substrate and function, it is no wonder that the members of the ABCA subfamily could be involved in several biological processes within the cells. Therefore, their role in promoting cancer development is investigated in several cancers including CRC, prostate, lung, osteosarcoma and renal cancer. Given the fact that they can transport signalling molecules and lipids, there is a high chance that they could impact cancer cells proliferation, resistance and migration. As mentioned earlier cholesterol dysregulation is linked to tumour development, hence proper regulation of its level is fundamental and the ABCA transporters (namely ABCA1, A8 and A12) are known to function in the transport of cholesterol [34]. Moreover, the transport of lipids besides cholesterol such as phospholipids and sphingolipids can affect other processes such as the regulation of immune cells and inflammation [35]. An interesting example of ABCA transporters and their involvement in immune signalling is ABCA1 and macrophage activation. Cancer cells can cause a decrease in membrane lipids of macrophages, resulting in increased activation of pathways that control the activation of macrophages and control the function of tumour-associated macrophages (TAMs) [27]. TAMs are strongly linked to tumour progression and invasion. In a study conducted by Goossens et al. [36] it was shown that targeting ABCA1 inhibits the function of TAMs and limits tumour progression. This provides an indication that using these lipids transporters as a target could lead to the development of a novel mechanism to target malignancies.

## 4. The Role of ATP-Binding Cassette Subfamily A in Colorectal Cancer

**Figure 2:**
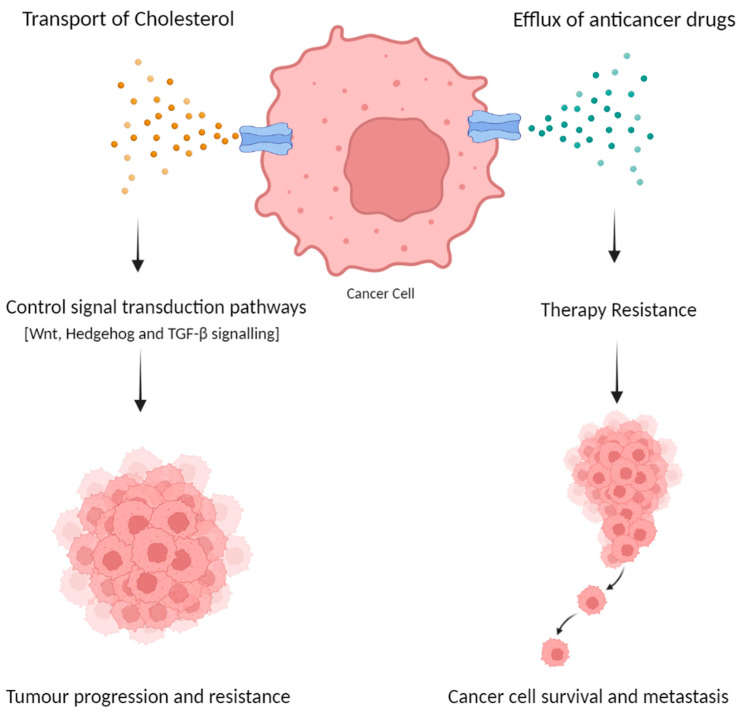
Proposed involvement of ABCA subfamily transporters in colorectal cancer progression and resistance. ABCA members can be overexpressed in cancer cells. They are mainly known for cholesterol transport, which can control various signalling pathways related to EMT and promote cancer progression and resistance. Additionally, ABCA transporters can promote drug efflux from the cells causing therapy resistance and leading to cancer cell survival. Figure generated by BioRender.

As the role of ABC transporters in the development of drug resistance and tumour development in cancer has been well investigated in many studies, the expression of ABCA transporters is seen to be altered in numerous types of cancer. These alterations can be at the mRNA or protein level and are usually linked to poor prognosis and survival. In CRC the use of chemotherapeutic agents such as 5-fluorouracil (5-FU), irinotecan and/or oxaliplatin remains an issue due to the development of drug resistance. Differential expression of ABC transporters has been observed in CRC and is considered one of the causes of therapy resistance and tumour progression [37]. One study demonstrated that anticancer drugs activate the IRE1α–XBP1 axis and induce the expression of ABCB1, ABCC1 and ABCG2, and targeting this pathway overcomes the drug resistance of CRC cells [38]. Furthermore, the expression of some of these transporters is linked to a better prognosis while others are associated with a worse prognosis. For instance, ABCC2 was shown to be linked to a better outcome in CRC patients [37]. In addition, ABCB4 could function as a tumour suppressor gene in CRC, as evidenced by the finding that 5-FU-resistant colon cancer cells exhibit downregulation of the ABCB4 gene [39]. On the other hand, ABCB1 was also found to modulate the resistance to apoptosis [40,41] and control cell proliferation in CRC, and the suppression of ABCB1 in a colon cancer cell line was demonstrated to inhibit proliferation by resulting in a G0/G1 phase cell cycle arrest in both in vitro and in vivo studies [42]. In comparison with other ABC transporters, the ABCA subfamily has a role in cancer, not only by promoting therapy resistance but also by potentially promoting tumour initiation and progression. A study conducted by Ohtsuki et al. [43] showed that undifferentiated colon cancer cell lines G-112 were found to express ABCA5 mRNA, opposed to normal colon tissues. The well-differentiated colon cancer cell line CX-1, in contrast, expressed ABCA2 mRNA. Therefore, ABCA5 induction could be associated with the differentiation state of human colon cancer and contribute to the growth of tumours. Furthermore, all ABCA transporters are involved in lipids, mainly cholesterol homeostasis, which, as previously stated in this review, can play a major part in cancer cell growth and proliferation [27]. Cholesterol metabolism is usually altered in CRC cancer, hence why these lipid transporters may be over-expressed in cancer cells where cholesterol is stored in large quantities, and the efflux of cholesterol out of the cells can act as signalling molecules as described earlier and activate pathways that favour tumour progression and resistance (Figure 2). Table 1 enlists the human ABC superfamily A transporter, their cellular localization, lipophilic substrate and possible role in colorectal cancer.

### 4.1. The Role of ABCA Subfamily in Colorectal Cancer Progression

In CRC, as the tumour progress, the tissue starts a transition from well-differentiated to poorly differentiated adenocarcinoma, in which cancer starts to grow and spread quickly. As stated previously, the mRNA expression of the ABCA5 transporter was reported to not be expressed in normal colon and elevated in poorly differentiated compared to well-differentiated colon adenocarcinoma [43]. Although the exact mechanism of action of ABCA5 in CRC is not confirmed, this study could indicate the possible role of ABCA5 in the progression of CRC cancer either by acting as a pump to efflux drugs out of the cells or, as another study mentioned, it could have a possible function in thyroid hormone homeostasis [44]; an example of that is thyroxine (T4), which can possibly promote the development of CRC [45]. As a cholesterol transporter, mRNA ABCA5 expression was induced in response to an increased cholesterol level [46]. This also could indicate its role in tumour progression as CRC cells show an increase in membrane lipid content to promote cell proliferation and development [23]. Another study by Aguirre-Portolés et al. [47] introduced the ATP-binding cassette transporter (ABCA1), a regulator of cholesterol transport, as a novel indicator of colon cancer invasion and survival. Their goal was to comprehend the significance of ABCA1 and the management of cholesterol transportation in the dissemination of CRC throughout the advanced stages of the disease, as well as its viability as a therapeutic target. Initially, they examined the mRNA expression in 71 patients with stage II CRC and 66 with stage III CRC patients to assess the significance of ABCA1 regulation in CRC staging. By using quantitative real-time PCR, they quantified the ABCA1 mRNA expression, and the results showed a considerable increment in stage III-CRC patients [47]. Collectively, these findings enable the notion that ABCA1 can be utilized as a potential novel marker whose expression levels could be employed as a predictive biomarker in CRC patients. The exact mechanism by which ABCA transporters promote tumour progression is not yet confirmed. One possible way could be explained with ABCA1, as in a study mentioned earlier [47] it was stated that ABCA1 could promote carcinogenesis by enhancing the development of tumours and their proliferation in addition to the activation of the epithelial–mesenchymal transition (EMT), which is a process where epithelial cells lose their characteristics and acquire migratory and invasive properties. This was confirmed by investigating the level of EMT markers, namely of E-cadherin (E-CAD) and vimentin (VIM) [47]. When cancer cells start to gain migratory properties cell surface proteins such as E-CAD are disassembled, and the cytoskeletal protein vimentin will replace the intermediate filament cytokeratin [48]. In that research, it was observed that cells that overexpress ABCA1 had a lower level of E-CAD and a higher level of VIM; this confirmed that ABCA1 could promote tumour progression by promoting EMT and cell migration [47].

### 4.2. Therapy Resistance

Drug resistance is considered one of the leading causes of death in cancer., A well-known role of ABC transporters in cancer is therapy resistance but the exact mechanism is not fully explained. These proteins act as pumps to extrude chemotherapeutic drugs from cancer cells. Therefore, inhibiting ABC transporters is a crucial strategy for overcoming drug resistance. For CRC, the development of treatments is becoming more complicated due to the resistance to various anti-cancer drugs. A study that investigated the effect of a compound that inhibited ABC transporters reported an increase in the mRNA expression of ABCA2, ABCA1 and mainly ABCA5 in chemoresistant CRC cell lines HCT116-FOr and H716 compared to chemosensitive HCT116 cells [49]. Since ABCA5 and ABCA2 are located in the membrane of the lysosome, they can promote drug resistance by storing the drug in the lysosome and promoting drug extrusion [27]. Furthermore, after treatment with a compound that sensitized chemoresistant CRC cells to anti-cancer drugs, it was observed that the overexpressed ABC transporters such as ABCA5 were downregulated [49]. Similarly, ABCA5 showed increased expression in the laryngeal Hep-2 cancer cell lines that became resistant to a higher dose of 5-fluorouracil, indicating the possible role of ABCA5 in cancer chemoresistance [50]. Moreover, as ABCA transporters are responsible for cholesterol transport, it was reported that cholesterol could be involved in MDR [51]. Alteration in cholesterol metabolism not only helps with the energy demand for cancer cells but also can control multiple signal transduction pathways that are linked to EMT such as Wnt, Hedgehog and TGF-β signalling to promote tumour aggressiveness and resistance, either by directly controlling the activity of protein intermediates or by regulating the lipid rafts. It was reported that EMT triggers MDR through drug efflux, enhancement, cell growth slowdown and apoptotic pathways evasion [51]. Other members of ABC transporters including ABCB1 and ABCC1 are targeted to resolve MDR in cancer; however, it is important to mention that none of these drugs is pharmacologically successful with some causing adverse side effects on the function of normal cells [27]. Nonetheless, limited data are available when it comes to targeting ABCA subfamily in CRC. A study that investigated imatinib resistance in chronic myeloid leukaemia utilized the drug Celecoxib to sensitize the cells to imatinib by inhibiting different ABC transporters including ABCA2 [52]. This provided some information about possibly targeting ABCA transporters in CRC to overcome drug resistance. Moreover, since ABCA transporters and cholesterol are associated with CRC, therapeutic agents targeting cholesterol uptake and influx are an excellent potential approach in CRC [27].

**Table 1:** Human ABC superfamily A transporter, their cellular localization, lipophilic substrate and possible role in colorectal cancer. (*) uniport database/www.uniprot.org, accessed on 11 December 2022 [33]; (**) https://doi.org/10.1016/j.bbamem.2016.09.023, accessed on 11 December 2022 [34].

Transporter	Cellular Localization (*)	Lipophilic Substrate (**)	Possible Role in CRC
ABCA1	Plasma membrane, lysosome, endoplasmic reticulum, Golgi apparatus	Cholesterol Phospholipids Sphingomyelin	Therapy resistance [49] Tumour progression [47]
ABCA2	Plasma membrane, lysosome, endosome	Cholesterol	Therapy resistance [49]
ABCA3	Plasma membrane	Cholesterol Phospholipids Sphingomyelin	-
ABCA4	Plasma membrane, endoplasmic reticulum	Phospholipids	-
ABCA5	Plasma membrane, lysosome, Golgi apparatus	Cholesterol	Therapy resistance [49] Tumour progression [43]
ABCA6	Golgi apparatus	*-*	-
ABCA7	Plasma membrane, endosome, lysosome, endoplasmic reticulum, Golgi apparatus	Phospholipids	-
ABCA8	Plasma membrane	Cholesterol	-
ABCA9	Plasma membrane	-	-
ABCA10	Plasma membrane	-	-
ABCA12	Plasma membrane, lysosome	Phospholipids	-
ABCA13	Plasma membrane	-	-

## 5. Clinical Implications and Future Directions

Emerging evidence indicates that ABCA transporters have crucial implications as diagnostic and prognostic biomarkers; their function in lipid homeostasis and their possible role in tumour progression and MDR highlight their potential use as diagnostic or therapeutic targets. Since altered lipid metabolism is confirmed as a potential cause of CRC, targeting these lipid transporters could be useful in detecting and treating CRC. The exact role of ABCA transporters in CRC is yet not confirmed; while many studies focus on the mRNA level, other studies should be conducted to assess the protein level of ABCA members in CRC since the protein level is reported to be higher than the mRNA level for some transporters [27]. Interestingly the overexpression of ABC transporters could be due to posttranscriptional regulations by microRNAs (miRNAs); therefore, miRNAs could be used to regulate the expression of ABCA transporters in CRC [11]. Utilizing the expression of these transporters in different stages of colorectal cancer tissue could provide information on their possible association with CRC progression and metastasis and the likely use of these transporters as novel markers. Additionally, since the expression of ABCA transporters varies in different types of cancer, their role could also differ depending on the substrate they transport and their cellular location, which requires further investigation. Analyzing the epigenetics mechanism that could modulate the expression of ABCA proteins in cancer might be helpful. Furthermore, since this study highlights the possible involvement of the ABCA subfamily in CRC, targeting these transporters either by an agent that blocks their activity or possibly by gene knock-out using genetic engineering methods could provide an understanding of their actual function in tumourigenesis. Finally, even with the limited data available, this review provides insight into the significance of the ABCA subfamily in CRC. Perhaps in the future, more studies will be focused on their expression and their role in tumour development, which can provide new approaches in targeting colorectal cancer.

## 6. Conclusions

The high incidence and mortality rate of CRC has made it a major public health issue on a global scale. Herein we have reviewed the role of ABC transporters, primarily the ABCA superfamily, in CRC. We currently only have a basic understanding of how ABCA transporters are involved in human diseases. Excluding substrates, the identification of therapeutic modulators/inhibitors remains the most difficult task. As evidenced by the fact that alteration in the expression of different ABCA transporters has been linked to numerous malignancies, they could play a crucial role in a variety of physiological systems. Lastly, since CRC is characterized by altered lipid metabolism and the transport of various lipid molecules depends heavily on the ABCA transporter, ABCA members might be potential candidates as novel biomarkers or therapeutic targets in CRC. Despite these various studies, the ATP-binding cassette subfamily A (ABCA) has not been the subject of many investigations. However, what was discussed within the scope of this review points to the critical importance of ABCA in colorectal cancer progression and drug resistance. In light of this, research into the processes by which ABCA transporters contribute to the development of colorectal cancer, therapy resistance and potential ABCA inhibitors, is essential.

### Limitations

The fundamental limitation of this review is the absence of prior research studies on the subject. The significance of ATP-binding cassette subfamily A (ABCA) in colorectal cancer progression, migration and drug resistance was not adequately investigated, as it is discussed in this paper. Additionally, more in-depth details concerning the ATP-binding cassette subfamilies, such as their molecular structure and the mechanisms by which they transform various molecules, could have been added.

## Data Availability

Not applicable.
